# Evaluation of Risk Factors Associated with Recurrence and Death in Patients with Thyroid Cancer From 2008 to 2023 in the West of Iran

**DOI:** 10.34172/jrhs.2024.167

**Published:** 2024-08-01

**Authors:** Salman Khazaei, Soheil Abdollahi Yeganeh, Seyed Ahmad Raza Salim Bahrami, Shiva Borzouei

**Affiliations:** ^1^Research Center for Health Sciences, Hamadan University of Medical Sciences, Hamadan, Iran; ^2^Department of Epidemiology, School of Public Health, Hamadan University of Medical Sciences, Hamadan, Iran; ^3^Neurosurgery Research Group, Student Research Committee, School of Medicine, Hamadan University of Medical Sciences, Hamadan, Iran; ^4^Department of Anesthesiology, School of Medicine, Hamadan University of Medical Sciences, Hamadan, Iran; ^5^Department of Endocrinology, School of Medicine, Hamadan University of Medical Sciences, Hamadan, Iran

**Keywords:** Treatment outcome, Recurrence, Mortality, Thyroid cancer

## Abstract

**Background:** Thyroid carcinoma (TC) is a global clinical concern, and its incidence has progressively increased worldwide. Early detection of TC and subsequently decreased age at the diagnosis seem to result from extensive employment of imaging modalities, biopsy techniques, and improvements in the healthcare system.

**Study Design:** A retrospective cohort study.

**Methods:** Overall, 400 patients diagnosed with TC following thyroidectomy in the Endocrinology Clinic, who were followed for fifteen years, were investigated in this study. The checklist included patients’ demographic characteristics, clinical information, and response to treatment, recurrence, and death.

**Results:** There were 19.25% men and 80.75% women. The mean age was 41.005±15.58 years. The risk of death and recurrence was significantly higher in men, patients>65 years, smokers, patients with a family history of TC, undifferentiated cancer, multifocality, and stages III and IV (*P*<0.001). Each additional year of life was associated with a 21% increase in the risk of death (*P*<0.001). Smoking was associated with a 4.36-fold increase in the risk of death (*P*=0.05). For each additional year of life, the probability of recurrence increased by 3% (*P*=0.009). Men were 4.73 times more likely to recur (*P*<0.001) than women.

**Conclusion:** To employ the proper therapeutic intervention and perform meticulous postoperative surveillance, it is crucial to consider the predictive influence of pertinent elements. Diagnosing TC in its early stages is essential for the healthcare system because of the increased incidence, younger age at diagnosis, and overall favorable prognosis of TC.

## Background

 The incidence of thyroid cancer (TC) has steadily increased worldwide over the past three decades, due to advancements in diagnostic methods.^1^ Women account for more than 75% of TC cases. This type of cancer is highly common in adults between the ages of 20 and 55 years.^2,3^

 Fortunately, TC can be treated, and 5-year and 10-year survival rates are 97% and 85–93%, respectively. The prognosis is significantly affected by age.^4^ Although several factors increase the risk of TC, including female gender, age, exposure to radiation, family history of TC, and a number of genetic factors, its exact etiology is unknown.^5^ Papillary carcinoma (80%–90%), follicular carcinoma (5%–10%), medullary carcinoma (3%–5%), anaplastic carcinoma (less than 5%), and others (1%–2%) are the subtypes of TC. It is highly important to know the subtypes of TC to determine the prognosis of the disease and the appropriate treatment.^6^ There are several stages and scores to determine the prognosis. The tumor, node, and metastasis staging approach is preferred by the American Joint Committee on Cancer.^7^ Risk assessment plays a highly important role in determining the type of treatment for patients.^8^ The prognostic guidelines published by the American Thyroid Association are currently the most accepted system for assessing the risk of recurrence and mortality of TC. These guidelines integrate a comprehensive array of clinicopathologic features to stratify patients into low- and intermediate-risk groups.^9^ Surgery is the primary treatment for TC. Treatments with iodine, chemotherapy, and target therapy are the next alternatives.^10^

 This study aimed to evaluate factors affecting therapeutic response, recurrence, and death rate in patients with TC in the west of Iran. Furthermore, we assessed recurrence and death rates on the American Thyroid Association risk assessment with other clinicopathologic factors.

## Methods

 All patients diagnosed with TC, confirmed by histopathological investigations following thyroidectomy, were enrolled in this retrospective cohort study and meticulously assessed from January 2008 to December 2023 in the specialized endocrine clinic in Hamadan, Iran. A comprehensive institutional database was used to identify patients, and those with missing data were excluded from the study. The patient’s demographic information, clinical findings, and 10-year follow-up data were collected using a checklist. Demographic information included gender, age, smoking status, and family history of TC in first-degree relatives. Clinical findings encompassed data on co-existing thyroid disorder, thyroid characteristics, cervical adenopathy, type of malignancy based on pathology results, unifocal and multifocal, stage group, risk staging, response to treatment, and follow-up data related to recurrence and death. The local ethical committee granted ethical approval for this project (IR.UMSHA.REC.1402.221).

 The staging was determined based on the tumor, node, and metastasis system, and the risk staging was determined based on the stage of the group and the age of the people.^5^ Response to treatment was determined based on the laboratory findings of thyroglobulin and antithyroglobulin and a whole-body scan.^6^

 Frequencies and percentages, as well as means and standard deviations, were used in the descriptive statistics section for qualitative and quantitative variables, respectively. The Chi-square test and Fisher’s exact test were utilized to investigate the relationship between death and recurrence with demographic and clinical information. The recurrence in patients with TC was predicted with logistic regression at a significance level of 0.05. Hosmer and Lemeshow’s approach was employed for model building, and variables with *P* values less than 0.2 in the crude model were entered into the multivariable model. The results of the logistic regression model were presented with an odds ratio (OR) and a 95% confidence interval (CI). All analyses were performed by Stata software, version 17.

## Results

###  Demographic features and clinical information

 The current study evaluated a total of 400 patients diagnosed with TC during 15-year and 10-year follow-ups. There were 77 (19.25%) men and 323 (80.75%) women, and their average age was 41.005 ± 15.58 years. Throughout the study period, a total of 32 cases (8%) resulted in death, while 116 cases (29%) experienced recurrence. Both death and recurrence rates were higher among men than women (23.38% vs. 4.33% and 59.74% vs. 21.67%, respectively; *P*< 0.001). More than half of the patients were between 25 and 45 years old (54%), while both death and recurrence risk rates were higher among patients older than 65 years old (*P*< 0.001).

 Based on the results, 53 (13.25%) patients were identified as smokers. The death rate and recurrence risk were both statistically higher in this group (*P*< 0.001). A small proportion of patients (8.25%) had a family history of TC. This item was associated with an increased risk of death (*P*= 0.024) and recurrence (*P*= 0.01).

 Based on the co-existing thyroid disorder, most participants were euthyroid (87.75%). There was no significant relationship between death (*P*= 0.960) or recurrence (*P*= 0.29) and co-existing thyroid disorders. It was revealed that the death rate was literally higher in patients with diffuse goiter (*P*< 0.001). Additionally, a significant connection was detected between multinodular goiter and recurrence risk (*P*= 0.017). A total of 289 patients (72.25%) did not exhibit cervical adenopathy. There was a relationship between bilateral cervical lymph node involvement and an increased risk of death and recurrence (*P*= 0.023 and *P*< 0.001, respectively). More details on this section are described in Table 1.

**Table 1:** Relationship between patients’ demographic information and clinical findings with the rate of death and recurrence

**Variables**	**Number**	**Death (n=32)**	**Pearson Chi**^2^	***P*** ** value**	**Recurrence (n=116)**	**Pearson χ**^2^	***P*** ** value**
Gender			30.63, df = 1	0.001		43.76, df = 1	0.001
Male	77	18			46		
Female	323	14			70		
Age group (y)			72.33, df = 3	0.001		21.87, df = 3	0.001
15-24.9	47	1			12		
25-44.9	216	5			48		
45-64.9	99	10			34		
65 +	38	16			22		
Smoking status			55.95, df = 1	0.001		40.70, df = 1	0.001
Yes	53	18			35		
No	347	14			81		
Family history of thyroid cancer			5.06, df = 1	0.024		6.63, df = 1	0.011
Yes	33	6			16		
No	367	26			100		
Co-existing thyroid disorder			1.91, df = 4	0.968		5.45, df = 4	0.293
Euthyroid	349	30			106		
Clinical hypothyroidism	12	0			2		
Subclinical hypothyroidism	14	1			5		
Clinical hyperthyroidism	20	1			3		
Subclinical hyperthyroidism	5	0			0		
Thyroid characteristics			22.64, df = 4	0.001		11.61, df = 4	0.017
Normal	7	0			2		
Diffuse goiter	12	5			2		
Single nodular goiter-right	146	6			30		
Single nodular goiter-left	90	7			27		
Multinodular goiter	145	14			55		
Cervical adenopathy			11.66, df = 6	0.023		158.65, df = 6	0.001
No	289	16			34		
Right	51	6			32		
Left	17	2			12		
Bilateral	33	6			28		
Behind the neck	2	0			2		
Bilateral and behind the neck	8	2			8		

*Note*. df: Degree of freedom.

###  Histopathology, risk stratification, and therapeutic response

 In this study, the most common types of TC were papillary TC (71.75%), micropapillary TC (12%), follicular TC (FTC, 7%), and Hurthle cell carcinoma (5%), respectively. Anaplastic TC (ATC), medullary TC, lymphoma, and poorly differentiated TC (PDTC) were observed in 1.25%, 1.75%, 0.75%, and 0.5% of participants, respectively. The death rate was 100% in patients with ATC, PDTC, and lymphoma (*P*< 0.001).

 In addition, 140 out of 400 patients (35.9%) had multifocal TC, while others (64.1%) had unifocal TC. Regarding a 12.14% death and 52.86% recurrence risk, a significant correlation was observed between multifocality and both death and recurrence risk rates (*P*< 0.001).

 The lowest and highest mortality rates were related to stage I (1.48%) and IVC (100%), respectively (*P*< 0.001). The highest risk of recurrence was 100% in stage III, and stage IVB and IVA patients had the next highest risk of recurrence at 91.67% and 80%, respectively (*P*< 0.001). High-risk patients had higher mortality and recurrence than those with intermediate- and low-risk rates (*P*< 0.001). On the other hand, recurrence risk was considerably increased in intermediate- and high-risk patients (*P*< 0.001). Regarding therapeutic response, the highest risk of recurrence and death was observed in structural incomplete division (*P*< 0.001, Table 2).

**Table 2:** Relationship between histopathological characteristics, tumor staging, risk stratification and response to treatment with mortality and recurrence

**Variables**	**Number**	**Death** **(n=32)**	**Pearson Chi**^2^	***P*** ** value**	**Recurrence** **(n=116)**	**Pearson χ**^2^	***P*** ** value**
Type of malignancy			130.94, df = 7	0.001		37.94, df = 7	0.001
Papillary	287	14			90		
Micropapillary	48	0			0		
Follicular	28	5			12		
Hurthle cell carcinoma	20	1			6		
Medullar	7	2			3		
Anaplastic	5	5			0		
Lymphoma	3	3			3		
Poorly differentiated thyroid carcinoma	2	2			2		
Focal			17.34, df = 1	0.001		63.84, df = 1	0.001
Uni focal	250	5			37		
Multi focal	140	17			74		
Stage group			168.07, df = 5	0.001		95.83, df = 5	0.001
I	337	5			65		
II	33	6			26		
III	4	1			4		
IVA	5	3			4		
IVB	12	8			11		
IVC	4	4			1		
Risk staging			79.61, df = 2	0.001		208.83, df = 2	0.001
Low risk	249	1			12		
Intermediate risk	102	7			64		
High risk	32	12			32		
Response to treatment			50.97, df = 3	0.001		309.47, df = 3	0.001
Excellent	208	0			1		
Biochemical incomplete	23	3			1		
Structural incomplete	9	17			89		
Indeterminate	61	0			7		

*Note*. df: Degree of freedom.

 The results of the multivariable logistic regression regarding the predictors of death in patients with thyroid tumors are summarized in Table 3. Based on the findings, after adjusting for other variables in the model, each additional year of age was associated with a 21% increase in the odds of death (OR = 1.21, 95% CI = 1.1, 1.34, *P*< 0.001). Smoking was associated with a 4.36-fold increased risk of death (*P*= 0.050). The odds of death in cancers with distant metastases (M1) were 7.9 times that of those without metastases (M0) (OR = 7.9, 95% CI = 1.39, 42.76, *P*= 0.032). Patients classified as moderate- and high-risk cases also had significantly higher odds of death compared to low-risk patients (*P*< 0.01).

**Table 3:** Predictors of death in patients with thyroid carcinoma based on logistic regression

**Variables**	**Adjusted OR (95% CI)**	***P*** ** value**
Age (y)	1.213 (1.110, 1.343)	0.001
Smoking		
No	1.000	
Yes	4.362 (1.000, 19.422)	0.052
Metastases		
M0	1.000	
M1	7.900 (1.393, 42.761)	0.032
Risk		
Low risk	1.000	
Intermediate risk	49.00 (3.352, 716.320)	0.004
High risk	214.0 (6.521, 705.091)	0.003

*Note*. OR: Odds ratio; CI: Confidence interval.

 The results of the multivariable logistic regression regarding the predictors of recurrence in patients with thyroid tumors are outlined in Table 4. After adjusting for other variables in the model, there was a 3% increase in the odds of recurrence for each additional year of age (OR = 1.034, 95% CI = 1.008, 1.06, *P*= 0.009). Men seemed to have 4.73-fold higher odds of recurrence compared to women (OR = 4.73, 95% CI = 2.025, 11.05, *P*< 0.001). Additionally, patients with euthyroidism had 13.29-fold higher odds of recurrence than patients with hyperthyroidism (*P*= 0.0330). The odds of recurrence were significantly higher in patients with T3 and T4 stages compared to those with the T1 stage (*P*< 0.01). Furthermore, patients with N1a and N1b stages had significantly higher odds of recurrence in comparison to patients with the N0 stage (*P*< 0.001).

**Table 4:** Predictors of recurrence in patients with thyroid carcinoma

**Variables**	**Adjusted OR (95% CI)**	***P*** ** value**
Age (y)	1.034 (1.008, 1.060)	0.009
Gender		
Female	1.000	
Male	4.731 (2.025, 11.053)	0.001
Thyroid disease		
Hyperthyroidism	1.000	
Euthyroid	13.288 (1.232, 143.371)	0.033
hypothyroidism	3.900 (0.273, 55.959)	0.320
Tumor		
T1	1.000	
T2	1.352 (0.453, 4.051)	0.591
T3	5.070 (1.741, 14.720)	0.003
T4	99.633 (9.112, 208, 862)	0.001
Nodes		
N0	1.000	
N1a	8.832 (2.763, 28.331)	0.001
N1b	26.721 (12.010, 59.432)	0.001

*Note*. OR: Odds ratio; CI: Confidence interval.

## Discussion

 This study was conducted to understand the potential significance of the mentioned factors in their predictive roles for the evolution of recurrence, death, and in patients with TC during the past 15 years. Several studies had evaluated different types of TC individually, even though there were papers assessing only DTC or non-DTC subtypes of TC alone. Our analysis consisted of all the main forms of TC to reach a comprehensive overview of this topic.

 Epidemiologic findings indicated that the TC incidence rate is higher among females than males. Perhaps it can be said that early referrals to treatment systems and earlier diagnosis using new methods are one of the reasons for the higher rate of thyroid cancer in females. By modulating both the enzymatic machinery of the thyrocyte and the inflammatory process linked to tumor growth, estrogens may promote the growth and invasion of tumoral cells and enhance the generation of mutagenic chemicals in the thyroid cell.^7^ In our study, the female/male (F/M) ratio was 4.2/1. However, it is noteworthy to mention that both death and recurrence rates were higher among men than women (23.38% vs. 4.33% and 59.74% vs. 21.67%, respectively). Similar to our findings, a study reported a F/M ratio of 5.5/1 in 312 patients with TC.^8^ Another study showed that there was a decreased survival rate relevant to the male gender in Portugal.^9^ A multicenter study in Spain also declared that the recurrence rate was associated with the male gender.^10^

 The majority of cases in this study were dedicated to the 25–45-year-old group (54%). Consistent with our results, the reported mean age of patients with TC was 40 years old in a recent retrospective cohort study.^8^ The increased use of imaging modalities, biopsy procedures, and the improvement of the healthcare system have led to the age of diagnosis. It was also revealed that each additional year of age was associated with a 21% increase in the odds of death and a 3% increase in recurrence odds. Of note, patients > 65 years old were significantly in danger of higher death and recurrence rates in our analysis. A recent study has demonstrated that there is a connection between the age of > 45 years old and raised death risk.^11^ Another study also revealed that increasing age is correlated with adverse outcomes.^12^

 In the current study, 8.25% of TC patients had a family history of TC. Mortality and recurrence risk rates were 2.6 and 1.8 times higher in this group, respectively. Similar to our findings, Yaghoubi et al found that 5.2% of TC patients had a family history of TC (*P* = 0.009), while the death or recurrence rate was not mentioned in their study.^13^

 Smoking and death were significantly correlated in our analysis. We found that the mortality rate was 33.96% and 4.03% among smokers and non-smokers, respectively, and smoking was associated with a 4.36-fold increased risk of death (*P* = 0.05). Out of 53 patients using cigarettes, only 10 were female. Considering that a great number of smokers were men in our study and the male gender itself was a poor prognostic factor, we cannot strongly declare that smoking may increase mortality in patients with TC, and further well-designed investigations are necessary in this regard. A recent meta-analysis has revealed that smoking may reduce TC occurrence, particularly DTCs.^14^ However, eligible data were not reported concerning cigarette-related mortality in TC.

 The death risk among patients with non-DTC, including ATC, PDTC, and lymphoma, was 100%. Among patients with DTC, FTC was associated with the highest death (17.86%) and recurrence (42.86%) risk. In line with our findings, ATC (100%) had the highest risk of mortality in a recent study.^8^

 Multifocality may be present in 18%–87% of TC cases.^15^ This factor seemed to be associated with more aggressive features and, of course, an increased recurrence rate.^16^ In our study, although most cases were unifocal (64.10%), multifocality significantly seemed to be relevant to both death and recurrence risk. Similar to our findings, one study revealed that multifocality was associated with aggressive features and a risk of metastasis.^11^ Another study reported that multifocal tumors were associated with an increased risk of recurrence and a poor prognosis.^17^

 Our study results suggested that the odds of recurrence are significantly higher in patients with T3 and T4 stages compared to those with the T1 stage (*P* < 0.01) and in patients with N1a and N1b stages compared to patients with the N0 stage (*P* < 0.001). Consistent with our findings, a single-center observational study in Taiwan showed a 3.9-fold higher rate of recurrence in patients with stages III and IV compared to those with stages I and II.^18^ Concurrently, other studies found a higher recurrence risk among patients with advanced stages.^19,20^

 Our findings revealed that among patients with DTC, the highest death and recurrence rates were both dedicated to structurally and biochemically incomplete responses to treatment, respectively (*P*< 0.001). The findings of a historical cohort study confirmed that both death and recurrence risk were higher in biochemical and structurally incomplete responses than others,^21^ which conforms to the findings of our study.

 Regarding the recurrence site in our study, there was mainly local involvement (65.49%). It is noteworthy to mention that of 39 patients with distant metastasis and cases who had both local and distant metastasis, 35 patients (89.74%) were diagnosed with DTC subtypes. Of note, all the patients who had distant recurrence alone (not local) were dedicated to the DTC group (100%), with FTC having the highest frequency rate (81.82%). Although female/male is a rare occurrence in DTCs, it has been documented to hurt patients’ survival.^22-24^ We also found that the odds of mortality in patients with distant metastases were 7.9 times higher (*P* = 0.03). Medas et al reported that 5.6% of patients with DTC had distant metastasis and stated that metastasis to lymph nodes was highly associated with recurrence.^25^ Consistent with our findings, another study declared that comparing FTC to papillary TC, there was a 2.57-fold increase in the frequency of metastatic disease.^11^ The variability of the thyroid tumors included in the analysis and the prospective influence of different therapeutic procedures, along with the lack of genetic and molecular assessment, were the limitations of our study. These limitations, however, highlight issues that need to be explored further. Another limitation of the study was that many patients did not refer and had incomplete information; therefore, they were not included in the study.

**Highlights**

- The rate of death and recurrence was significantly higher in undifferentiated cancers, multifocality, and high stages.
- The rate of death and recurrence was significantly higher in men, the elderly, and smokers.
- The incidence rate of thyroid carcinoma was higher among women than men.
- The death risk among patients with non-differentiated thyroid carcinoma was 100%.

## Conclusion

 The rates of death and recurrence were significantly higher in men, the elderly, smokers, patients with a family history of TC, undifferentiated cancers, multifocality, and high stages. Considering the predictive impact of relevant factors, it is truly important to apply the appropriate therapeutic intervention and postoperative surveillance.

## Acknowledgements

 This study was approved by the Research Vice-Chancellor of Hamadan University of Medical Sciences (140203302483). The authors would like to express their thanks.

## Authors’ Contribution

**Conceptualization:** Shiva Borzouei.

**Data curation:** Shiva Borzouei and Soheil Abdollahi Yeganeh.

**Formal analysis:** Salman Khazaei.

**Funding acquisition: **Shiva Borzouei.

**Investigation:** Shiva Borzouei, Salman Khazaei, Seyed Ahmad Raza Salim Bahrami, and Soheil Abdollahi Yeganeh.

**Methodology:** Shiva Borzouei, Salman Khazaei, and Seyed Ahmad Raza Salim Bahrami.

**Project administration:** Shiva Borzouei.

**Resources:** Shiva Borzouei.

**Software:** Salman Khazaei.

**Supervision:** Shiva Borzouei.

**Validation:** Shiva Borzouei and Salman Khazaei.

**Visualization:** Shiva Borzouei and Soheil Abdollahi Yeganeh.

**Writing–original draft:** Shiva Borzouei, Salman Khazaei, Seyed Ahmad Raza Salim Bahrami, and Soheil Abdollahi Yeganeh.

**Writing–review & editing:** Shiva Borzouei, Salman Khazaei, Seyed Ahmad Raza Salim Bahrami, and Soheil Abdollahi Yeganeh.

## Competing Interests

 All authors certify that they are not affiliated to or involved in any organization or entity with any financial (e.g., honoraria, educational grants, participation in speakers’ bureaus, membership, employment, consultancies, stock ownership, or other equity interest, and expert testimony or patent-licensing arrangements) or non-financial (e.g., personal or professional relationships, affiliations, knowledge, or beliefs) interests in the subject matter or materials discussed in this manuscript.

## Ethical Approval

 The local ethical committee of Hamadan University of Medical Sciences granted ethical approval for this project (IR.UMSHA.REC.1402.221).

## Funding

 This study received financial support from Hamadan University Medical Sciences.
